# Comparison of data‐acquisition methods for the identification and quantification of histone post‐translational modifications on a Q Exactive HF hybrid quadrupole Orbitrap mass spectrometer

**DOI:** 10.1002/rcm.8401

**Published:** 2019-04-26

**Authors:** Joby Cole, Eleanor J. Hanson, David C. James, David H. Dockrell, Mark J. Dickman

**Affiliations:** ^1^ Department of Infection, Immunity and Cardiovascular Diseases University of Sheffield UK; ^2^ Department of Chemical and Biological Engineering University of Sheffield UK

## Abstract

**Rationale:**

Histone post‐translational modifications (PTMs) play key roles in regulating eukaryotic gene expression. Mass spectrometry (MS) has emerged as a powerful method to characterize and quantify histone PTMs as it allows unbiased identification and quantification of multiple histone PTMs including combinations of the modifications present.

**Methods:**

In this study we compared a range of data‐acquisition methods for the identification and quantification of the histone PTMs using a Q Exactive HF Orbitrap. We compared three different data‐dependent analysis (DDA) methods with MS2 resolutions of 120K, 60K, 30K. We also compared a range of data‐independent analysis (DIA) methods using MS2 isolation windows of 20 *m/z* and DIAvw to identify and quantify histone PTMs in Chinese hamster ovary (CHO) cells.

**Results:**

The increased number of MS2 scans afforded by the lower resolution methods resulted in a higher number of queries, peptide sequence matches (PSMs) and a higher number of peptide proteoforms identified with a Mascot Ion score greater than 46. No difference in the proportion of peptide proteoforms with Delta scores >17 was observed. Lower coefficients of variation (CVs) were obtained in the DIA MS1 60 K MS2 30 K 20 *m/z* isolation windows compared with the other data‐acquisition methods.

**Conclusions:**

We observed that DIA which offers advantages in flexibility and identification of isobaric peptide proteoforms performs as well as DDA in the analysis of histone PTMs. We were able to identify 71 modified histone peptides for histone H3 and H4 and quantified 64 across each of the different acquisition methods.

## 1 INTRODUCTION

Regulation of eukaryotic gene transcription is a complex, carefully orchestrated series of molecular interactions where epigenetic mechanisms of control are becoming increasingly recognized.^1^ In eukaryotes, a 147 bp length of DNA is coiled around a histone octamer (composed of H3 and H4 proteins and two H2A/H2B dimers) which forms a nucleosome; with the addition of H1 and regions of linker DNA this in turn forms chromatin.^2^ The chemical modifications of the N‐terminal tail of histones, termed post‐translational modifications (PTMs), alter the conformation of the chromatin thereby affecting the availability of the DNA^3^ to transcription factors.^1^ Therefore, these histone PTMs play key roles in regulating eukaryotic gene expression. Histone PTMs are laid down in a dynamic fashion and enzymatic activities exist that deposit and remove particular PTMs. Histone N‐terminal tails are the targets for PTMs since they protrude from the nucleosome and can make contact with adjacent nucleosomes, thus providing a mechanism for regulating regional protein–DNA and protein–protein interactions.^1^ In addition, the PTMs of histones provide binding sites for a number of effector molecules that can establish and orchestrate downstream events such as gene transcription. Therefore, these histone marks not only dictate chromatin structure but they also control access to the underlying DNA and hence are involved in all DNA‐based processes including gene expression.

Mass spectrometry (MS) has emerged as a powerful method to characterize and quantify histone PTMs as it allows unbiased identification and quantification of multiple histone PTMs, including combinations, in a single analysis. Recently, a plethora of different approaches have been described for the study of histone PTMs.^4^, ^5^ These include top‐down,^6^, ^7^ middle‐down^8^, ^9^ and bottom‐up approaches.^10^, ^11^ The top‐down approach provides information at the protein level, enabling the study of histone protein proteoforms and their associated combination of PTMs. The bottom‐up approach provides information at the peptide level, and provides information on histone peptide proteoforms. Different data‐acquisition strategies have been developed and employed for the bottom‐up analysis of histone PTMs. Data‐Dependent Acquisition (DDA) is the most commonly used and does not require any prior knowledge of the PTMs.^10^ During MS acquisition, the top N eluting peptides in terms of spectral peak intensity are selected for fragmentation and product ion analysis (MS/MS). However, the quantification of isobaric co‐eluting peptides using this approach proves challenging. In addition, low‐abundance modified peptides may not be selected for MS/MS and consequently not identified and quantified.

In light of this, Selective Reaction Monitoring (SRM) and Parallel Reaction Monitoring (PRM) methods have been developed.^12^, ^13^, ^14^ These approaches rely on the establishment of an inclusion list for all of the different peptide proteoforms to target for MS/MS. These are then monitored throughout the high‐performance liquid chromatography (HPLC) gradient and selected for MS/MS when detected. These targeted methods improve the sensitivity, especially for low‐abundance modified peptides, but are constrained by total cycle time for multiple PTMs as these can ionize in different charge states necessitating multiple entries in the inclusion list for a single species. They are limited by the number of transitions that can be monitored throughout the gradient and the need for prior knowledge of which ones to target. Moreover, once acquisition is complete, retrospective analysis for novel PTMs is not possible.

In order to overcome these limitations Data‐Independent Acquisition (DIA) methods have been gaining in popularity for discovery proteomics and are particularly suited to the study of PTMs.^15^, ^16^, ^17^, ^18^ A number of different DIA methods have been used to analyze histone PTMs. One of the first methods developed was SWATH™ (AB Sciex) designed for the triple time‐of‐flight (TOF) instruments. This method was successfully used to identify and quantify histone PTMs^17^ and involves a series of 85 isolation windows of variable sizes spanning the *m/z* range in which histone PTMs are found (see Table S1, supporting information). Subsequently, Krautkramer et al used a DIA method with regular 10 *m/z* isolation windows to identify and quantify the changes in histone PTMs following histone deacetylase inhibitor treatment.^18^ Using this approach enabled greater reproducibility than conventional DDA with consistently high numbers of histone peptides identified and with lower coefficients of variation (CVs) in relative abundance. Indeed, both the SWATH™ and other DIA methods were able to detect low‐abundance peptides. A previous study has shown that DIA protocols can also be adapted to lower resolution ion trap instruments.^15^ In this study, the authors demonstrated the adaptability of low‐resolution DIA to accurately identify histone PTMs in mouse embryonic stem cells. They compared a range of sequential isolation windows from 20 to 50 *m/z* on an LTQ‐Orbitrap. Furthermore, the same group had previously compared both a high‐resolution LTQ‐Orbitrap with a low‐resolution LTQ Velos Pro instrument for the analysis of histone PTMs in DDA mode using heavy isotope labeled synthetic peptides.^19^

In this study we compare a range of DDA and DIA methods for the identification and quantification of the histone PTMs using a Q Exactive HF hydrid quadrupole‐Orbitrap mass spectrometer. We analyzed histone PTMs in Chinese Hamster Ovary (CHO) cells, an important biological system for the production of biopharmaceuticals for over 25 years.^20^ CHO cells have the ability to grow in serum‐free media, achieving high yields and, furthermore, create human‐like PTMs. Despite their prevalence in industry the epigenetics of CHO cells have not been widely studied. We have examined the histone PTMs of a CHO‐S line that expresses an anti‐HER2 like IgG1 antibody and the changes in relative abundance of histone PTMs between days 2 and 4 of culture.

## 2 EXPERIMENTAL

### 2.1 Cell culture

CHO‐S cells were obtained from Cobra Biologics. They were grown in CD‐CHO supplemented with 8 mM L‐glutamine, 12.5 μg/mL puromycin and HT supplement (ThermoFisher Scientific) media for either 2 or 4 days. Then they were washed in phosphate‐buffered saline (PBS) and pelleted by centrifugation at 200 *g*.

### 2.2 Histone extraction and digestion

Histones were extracted following the protocol previously described in Minshull et al.^11^ Briefly, cell pellets underwent hypotonic lysis followed by acid extraction.^21^ Histones were re‐suspended in 100 mM of ammonium bicarbonate pH 8.0 before two rounds of chemical derivatization using propionic anhydride in isopropanol (1:3 ratio) for 15 min at 37°C, followed by trypsin digestion overnight and a further two rounds of derivatization.^18^ The samples were desalted using HyperSep hypercarb tips (ThermoFisher Scientific), prior to nano‐flow LC/ESI‐MS on a Q Exactive HF Orbitrap mass spectrometer (ThermoFisher Scientific).

### 2.3 LC/MS/MS methods

Samples re‐suspended in 0.1% trifluoroacetic acid (TFA) were analyzed on an Ultimate 3000 online nano‐LC system with a PepMap300 C18 trapping column (ThermoFisher Scientific), coupled to a Q Exactive HF Orbitrap (ThermoFisher Scientific). Peptides were eluted onto a 50 cm × 75 μm Easy‐spray PepMap C18 analytical column at 35°C. Peptides were eluted at a flow rate of 300 nL/min using a gradient of 3% to 25% over 55 min then 25% to 60% until 81 min. Solvents were composed of 0.1% formic acid (FA) and either 3% acetonitrile (ACN) (solvent A) or 80% ACN (solvent B). The loading solvent was 0.1% TFA and 3% ACN.

Data acquisition was performed in a number of different modes (as summarized in Table 1). DDA was performed in full scan positive mode, scanning 375 to 1500 *m/z*, with an MS1 resolution of 120,000, Automatic Gain Control (AGC) target of 1 × 10^6^ and a maximum fill time of 450 ms. The top 10 most intense ions from each MS1 scan were selected for collision‐induced dissociation (CID). MS2 resolution was set at either 120,000 (DDA120), 60,000 (DDA60) or 30,000 (DDA30) with the AGC target of 1 × 10^5^ and maximum fill times of 450, 220 and 100 ms, respectively, with an isolation window of 2 *m/z* and scanning range of 200–2000 *m/z*, normalized collision energy (NCE) 27. DIA was performed with three different settings. First, DIA60 had a full scan at a resolution 60,000, AGC target of 3 × 10^6^, maximum fill time of 55 ms, scanning range of 300 to 900 *m/z*; followed by 10 DIA windows at a resolution of 30,000, AGC target of 1 × 10^6^, isolation window of 20 *m/z* and NCE 26 for DIA60. DIA30 had full scan resolution of 30,000, AGC target of 3 × 10^6^, maximum fill time 100 ms, scanning range of 300 to 900 *m/z*; followed by 10 DIA windows at a resolution of 15,000, AGC target 1 × 10^6^, isolation window of 20 *m/z*, NCE 26. For DIA60 and DIA30 the isolation lists were calculated with the aid of Skyline and are summarized in Table S2 (supporting information).

**Table 1:** Main characteristics of the different data‐acquisition methods used in this study

		**Data‐dependent acquisition**			**Data‐independent acquisition**		
		**DDA120**	**DDA60**	**DDA30**	**DIA60**	**DIA30**	**DIAvw**
**MS1**	Resolution	120,000	60,000	30,000	60,000	30,000	30,000
	AGC	1.0E+06	1.0E+06	1.0E+06	3.0E+06	3.0E+06	3.0E+06
	Fill time (ms)	450	450	450	55	100	100
	Scan range (*m/z*)	375–1500	375–1500	375–1500	300–900*	300–900	300–900
**MS2**	Resolution	120 000	60 000	30 000	30 000	15 000	15 000
	AGC	1.0E+05	1.0E+05	1.0E+05	1.0E+06	1.0E+06	1.0E+06
	Fill time (ms)	450	220	100	Automatic	Automatic	115
	Loop count	10	10	10	10	10	85
	Isolation window	2	2	2	20 *m/z*	20 *m/z*	Variable
	NCE	27	27	27	26	26	26
	Scan range (*m/z*)				300–900	300–900	300–900

Finally, DIA variable window (DIAvw) had full scan resolution of 30,000, AGC target of 3 × 10^6^, maximum fill time 100 ms, scanning range of 300 to 900 *m/z*; followed by 85 DIA windows at a resolution of 15 000, AGC target 1 × 10^6^, maximum fill time 115 ms, with an isolation window scheme which varied to resemble SWATH™ (AB Sciex) (the variable isolation windows are summarized in Table S1, supporting information), and NCE 26. Characteristics of each run were established using RAWMeat (version 2.1, VAST Scientific).

### 2.4 Data analysis

RAW files were converted into MGF using MSConvert (ProteoWizard) for DDA runs. Searches were performed using Mascot Daemon 2.5.0 (using CHO Uniprot 10029 (downloaded 07/06/2017)), Arg‐C digestion, peptide tolerance 10 ppm, MS/MS tolerance 0.01 Da, no missed cleavages, peptide charges of 2, 3 and 4+, Fixed modifications (propionyl (K) and propionyl (N‐term)) and variable modifications (acetyl (K), methylpropionyl (K), dimethyl (K) and trimethyl (K)). FDRs were set to less than 2%. Searches were also performed in MS Amanda version 2.0.0.9695 (using CHO Uniprot 10029 (downloaded 07/06/2017), Arg‐C digestion, MS1 tolerance 10 ppm, MS2 tolerance 0.02 Da, no missed cleavages, Peptide charges of 2, 3 and 4+; fixed modifications (propionyl (N‐term)) and variable modifications (propionyl (K), acetyl (K), methylpropionyl (K), dimethyl (K) and trimethyl (K)).

Analysis for the efficacy of each search was performed on nine peptides covering H3 and H4. For these peptide proteoforms identified by Mascot or MS Amanda searches the top scoring ID of each peptide proteoform was recorded as was the number of different peptide proteoforms identified. Incompletely propionylated peptides were excluded.

Relative abundance was calculated using Skyline^27^ to first extract chromatographic peak areas for each peptide proteoform which was then normalized to the sum of the peak areas of all forms of that peptide. For DIA PTM identification was performed in Skyline (using prior knowledge of elution profile, dotIP >0.90 and <5 ppm).^28^ The relative abundances of histone PTMs and identification were also determined using EpiProfile 2.0.^22^ Statistics were calculated using GraphPad Prism version 7.03 (GraphPad Software).

## 3 RESULTS AND DISCUSSION

To study the effect of MS2 scan resolution on the identification and quantification of histone PTMs we compared three different DDA methods with MS2 resolutions of 30,000, 60,000 and 120,000. We also compared a range of DIA methods using MS2 isolation windows of 20 *m/z* at a resolution of 30,000 or 15,000 and DIAvw and compared these with the DDA methods.

## 4 DATA‐ACQUISITION METHODS

The baseline characteristics of each data‐acquisition method were established by initially calculating the duty cycles for each method as summarized in Figure S1A (supporting information). The higher the resolution, the longer the Orbitrap scan time, resulting in a longer duty cycle. This time was kept below 5 s for the DDA mode allowing at least 7 MS1 scans in a 30 s peak width, which is typical for peptides during chromatographic separations employed in this study. The DIAvw had the longest duty cycle of 5.1 s and the shortest was 1.4 s with DDA30. We examined the numbers of MS1 and MS2 scans for each method used. As expected, the lower resolution methods were associated with increased numbers of MS1 scans (Figure S1B, supporting information). A higher number of MS2 scans was obtained in DIA mode (Figure S1C, supporting information).

### 4.1 Identification of histone PTMs using DDA methods

**Figure 1:**
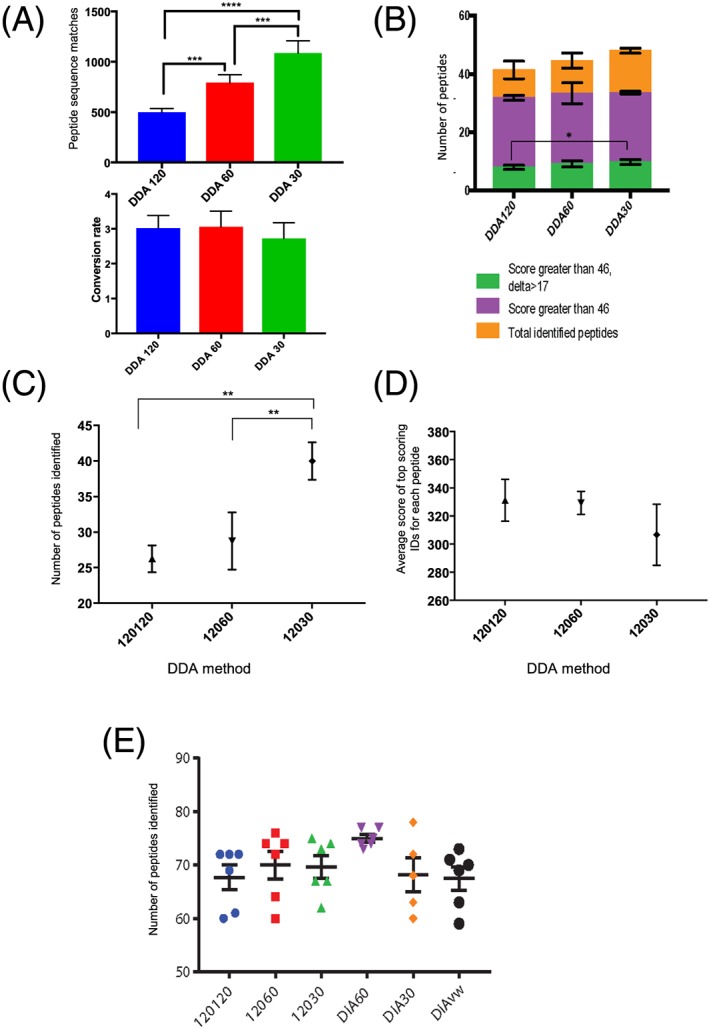
Identification of histone PTMs using different data‐acquisition methods with Mascot or MS Amanda. A, PSMs above the identity threshold and spectral utilization for peptide proteoforms identified using Mascot across the different DDA methods. Peptides from H3.1 and H4 were examined. One‐way analysis of variance (ANOVA) showed a significant difference between the number of PSMs (adjusted *p* <0.001) but no significant differences in conversion rates between each data‐acquisition method. B, The peptides with Mascot ion scores >46 and Delta scores >17 are represented. One‐way ANOVA showed a significant difference between the number of peptides with Mascot ion scores >46 and Delta scores >17 when comparing the DDA120 to DDA30 (*p* <0.02) (A to B, *n* = 5, illustrated are the mean and error bar = 1 standard deviation). C, Number of different peptides identified by MS Amanda search. One‐way ANOVA identified a significant difference between the number of peptides identified between the DDA30 and the other two methods (adjusted *p* <0.001). D, Average top score of each different peptide identified by MS Amanda search. (C to D, *n* = 4, illustrated are the mean and error bar = 1 standard deviation). E, Number of peptides identified using EpiProfile 2.0 in each different data‐acquisition method illustrates that there was slightly higher numbers of peptides identified with the DIA 60 approach (*n* = 6, illustrated are the mean and error bar = 1 standard deviation) [Color figure can be viewed at wileyonlinelibrary.com]

The numbers of different histone peptides identified was compared across the three different DDA acquisitions methods. Post‐acquisition data processing was performed using Mascot and the results are summarized in Figure 1. We initially assessed the number of peptide sequence matches (PSMs) and total number of queries in each of the experiments. The lower resolution scans were associated with a greater number of queries and consequently a greater number of PSMs (Figure 1A). However, the spectral utilization, the proportion of PSMs to the number of queries, was slightly lower in the DDA30.

To determine the accuracy of these potential identifications, we examined the Mascot ion scores associated with each peptide proteoform. The results show that as the MS2 resolution decreased, more scans were performed and more peptide proteoforms were identified (see Figure 1B). The highest Mascot peptide ion score, and therefore the most confident identification, was the same across all three methods despite the increasing ppm error in the lower resolution scans (Figure 1B). The proportion of peptides identified with Mascot ion scores greater than 46 (5% confidence threshold) was highest in the 120,000 resolution MS1 scans (77%, 75% and 70%, respectively).

Correctly identifying the position of PTMs can be challenging given that histone peptides are heavily modified and the near isobaric nature of acetylation and trimethylation. In order to further disambiguate the position of PTMs we looked at the Mascot Delta score for each of the peptide proteoforms identified across the different methods. Previous work in the field of phosphoproteomics has determined that a Mascot Delta score of greater than 17 was associated with accurate location of phosphorylation.^23^ Mascot Delta scores were calculated by taking the difference between the highest ion score for a given peptide and the score for the next possible peptide. Despite identifying a greater number of total peptides, the lower resolution scans did not do so with the same degree of confidence. The higher resolution scans had a higher proportion of peptides with a Mascot ion score greater than 46. However, the proportion of peptides with a Mascot Delta score of greater than 17 was the same in all three data‐acquisition methods (approximately 20%) (Figure 1B). The increased number of MS2 scans afforded by the lower resolution method resulted in a higher number of queries, PSMs and a higher number of peptide proteoforms with a Mascot ion score greater than 46 with no difference in the proportion of peptide proteoforms with Delta scores >17.

In addition to processing the data with Mascot, an alternative search engine, MS Amanda, was also used. MS Amanda places an emphasis on high‐accuracy MS2 data and is therefore optimized for high resolution and mass accuracy at both the MS1 and MS2 levels.^24^ As the MS2 resolution increased from 30,000 to 60,000 fewer peptides were identified (40, 29, 26, respectively) (see Figure 1C). The average top score of the peptides did not significantly increase as the resolution increased (see Figure 1D).

In summary these results indicate that no significant benefit is gained by performing DDA analysis using high‐resolution MS2 scans on the Q Exactive HF Orbitrap for the analysis of histone PTMs.

### 4.2 Identification of histone PTMs using DIA methods

Having examined the ability of the different DDA methods to identify histone PTMs, we then extended the comparison to different DIA methods. For the identification of histone peptides using DIA methods, data analysis was performed using EpiProfile 2.0 which was specifically developed for the identification and quantification of histone PTMs, and can process both DDA and DIA data.^22^, ^25^ We compared the total number of peptide proteoforms identified across all of the different acquisition methods for histones H3 and H4 (see Figure 1E). The results showed that on average 69 histone peptides proteoforms were identified in each method (ranging from 60 to 77). Forty‐seven peptides were identified in all of the runs (68% of average identified) and 90% of all the peptides were identified in at least 3 out of 6 runs in each method. These results show that the DIA60 method identified the most peptides, 75 across all 6 runs, of which 96% were identified in at least 3 runs.

### 4.3 Quantification of histone PTMs using DIA and DDA methods

Having established that all of the DDA and DIA methods were able to correctly identify the majority of the lysine methylation and acetylation PTMs on histones H3 and H4 we then turned our attention to the relative quantification of histone PTMs using the different acquisition methods. The relative abundance of each histone peptide proteoform was calculated as described above using both Skyline and EpiProfile.

**Figure 2:**
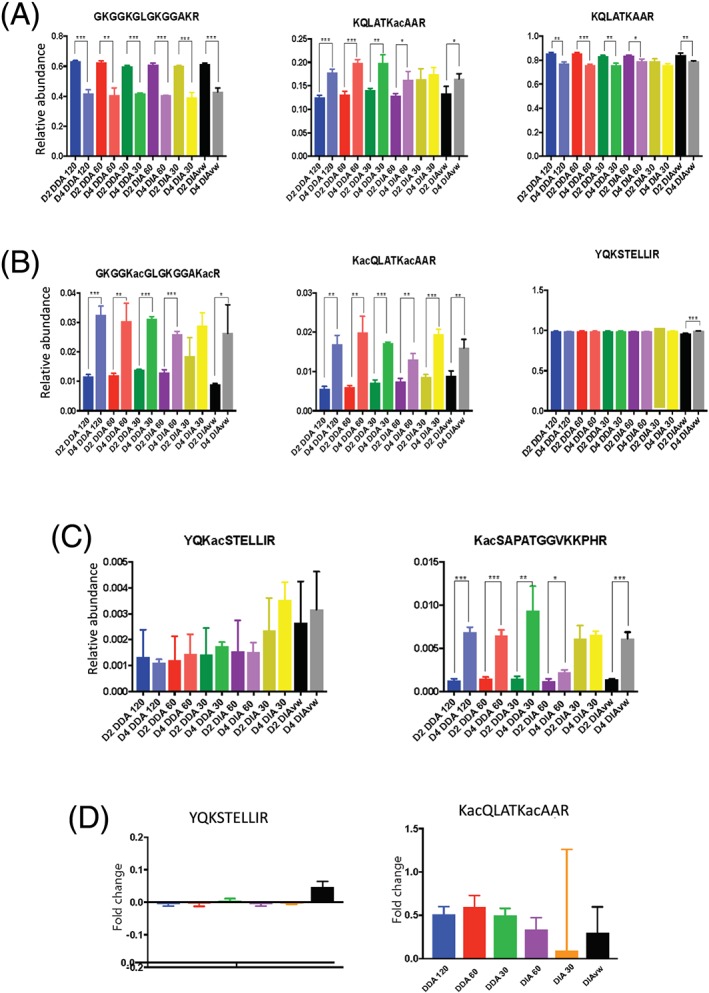
Comparison of relative abundance of histone PTMs between data‐acquisition methods. The relative abundances of histone PTMs for day 2 and day 4 CHO cells were calculated using EpiProfile 2.0 for the different data‐acquisition methods. A, Histone peptide proteoforms with high MS1 intensity GKGGKGLGKGGAKR on histone H4, KQLATKacAAR and the unmodified form on histone H3. B, Three peptide proteoforms with medium MS1 signal intensities. GKGGKacGLGKGGKacR and KacQLATKacAAR represent lower relative abundance peptide proteoforms for the respective peptides and YQSTELLIR represents high relative abundance. C, Peptide proteoforms of both low relative abundance and low MS1 intensity. D. Fold change between the relative abundance of PTM for day 2 and day 4 for the peptide YQSTELLIR and the peptide KacQLATKacAAR (two‐tailed unpaired t test, * *p* <0.05, ** *p* <0.01, *** *p* <0.001). The 95% confidence intervals from the unpaired t tests are shown in Table S3 (supporting information) [Color figure can be viewed at wileyonlinelibrary.com]

In order to compare the accuracy of relative abundance quantifications between each data‐acquisition method, we evaluated the ability to identify changes in the relative abundance of histone PTMs of CHO cells between day 2 of culture and day 4 (Figure 2) as these have been previously shown to alter over time in culture.^26^ In order to further analyze the quantitative differences obtained across these methods we focused on a number of peptide proteoforms that were initially identified as changing in abundance between days 2 and 4 of CHO cell culture.

As the overall ion intensity of a peptide may influence the ability of both correct identification and therefore quantification, we looked at examples of histone peptides with high, medium and low ion intensities which we defined as chromatographic peak heights of >9 × 10^9^, >8 × 10^7^, >3 × 10^5^ counts, respectively. The selected histone H3 and H4 peptide proteoforms also covered a range of relative abundances (0.5 to 99%).

#### 4.3.1 High‐intensity PTMs

Figure 2A shows that each method was able to confidently (*p* <0.01) report the change in relative abundance of the highly abundant histone H4 peptide GKGGKGLGKGGAKR between days 2 and 4. We next looked at the ability to correctly identify changes in the relative abundance of acetylation on K23 of H3 (KQLATKacAAR). In this case relative quantification is more challenging owing to the co‐elution of isobaric peptides (acetylation on K18 or K23). Therefore, the relative abundance was derived from the proportion of diagnostic y and b ions in the MS2 spectrum.^25^ As shown in Figure 2A, all of the different methods apart from DIA30 reported the change in relative abundance of the peptide proteoform. However, it should be noted that the DIA30 analysis showed the same trend in increase in K23 acetylation with reciprocal decrease in the unmodified form, but with p‐value = 0.164.

#### 4.3.2 Mid‐intensity PTMs

Further analysis of lower intensity peptides such as the dual acetylated peptide GKGGKacGLGKGGAKacR of H4 or the peptide KacQLATKacAAR of H3 is shown in Figure 2B. The results show that the difference in relative abundance between days 2 and day 4 samples was again observed across all methods with *p* <0.05 except in DIA30. Also of interest was the relative abundance of a peptide in which we did not expect to see a change between day 2 and 4. The DIAvw method showed greater variability (1.79% CV) in the relative abundance of the unmodified YQSTELLIR peptide from H3 compared with the other data‐acquisition methods.

#### 4.3.3 Low‐intensity PTMs

Finally, in the low‐intensity peptides such as YQKacSTELLIR on histone H3 (see Figure 2C), we observed no significant differences between the methods. When we examined the changes in the relative abundance of KacSAPATGGVKKPHR (H3K27ac) between days 2 and 4, we were able to detect the increase in acetylation in all three DDA methods and in both DIA60 and DIAvw, but not in DIA30.

In summary the results show that the data‐acquisition methods were all able to identify the same trend in the relative abundance of the more prominent PTMs (Figure 2D). However, both the DIA30 and DIAvw that had lower resolution and greater cycle time in the case of DIAvw appeared to not have the same degree of precision as the other methods.

### 4.4 Repeatability of the relative abundance quantification

**Figure 3:**
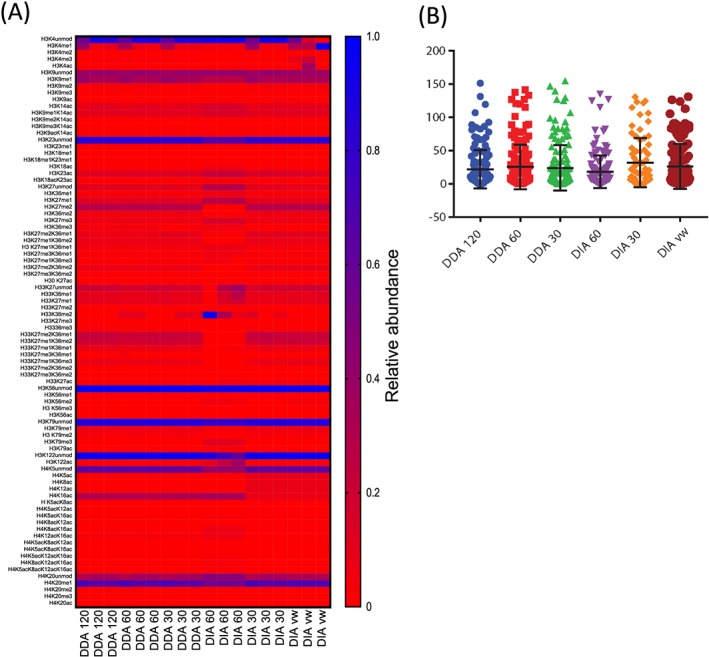
Relative abundance of histone peptides: A, Heatmap of the histone PTMs identified from CHO cells. The relative abundance of each peptide proteoform from three technical repeats identified in the different data‐acquisition methods is shown. B, Coefficients of variation (CVs) were calculated for all of the peptide proteoforms in each of the different data‐acquisition methods for both day 2 and day 4 samples (*n* = 5, error bars represent mean and standard deviation) [Color figure can be viewed at wileyonlinelibrary.com]

**Figure 4:**
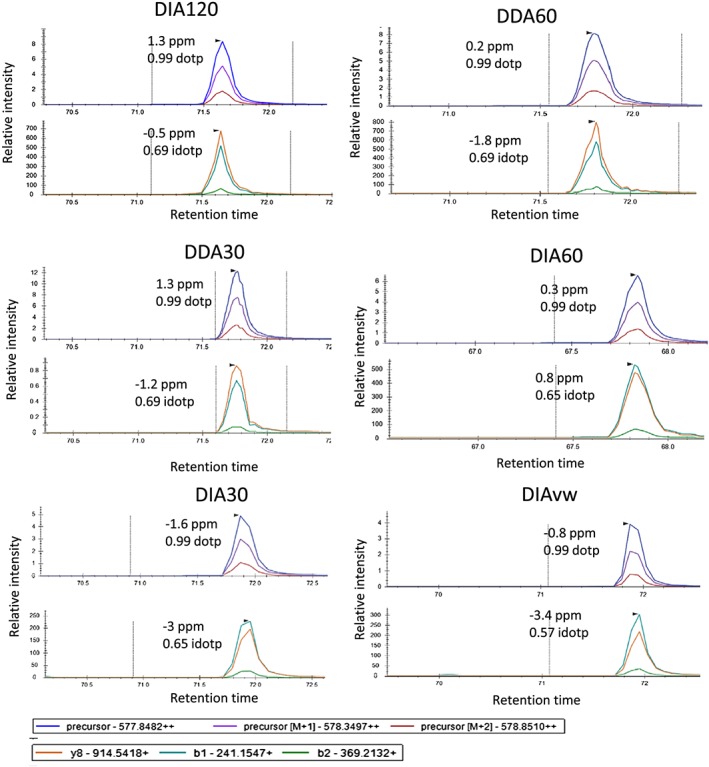
Representative extracted ion chromatograms for the peptide KQLATKAAR in each data‐acquisition method with ppm error for MS1 and MS2 spectra and the dotp (isotope dot product which is a comparison between the observed and theoretical isotope distributions) and idotp (dot product which compares the observed spectra and spectral library match) scores. This shows that they are of similar quality [Color figure can be viewed at wileyonlinelibrary.com]

In order to assess the repeatability of the relative abundance measurements of each acquisition methods, we examined three technical replicates for day 2 and day 4 and calculated the CVs for each peptide proteoform identified in all replicates (see Figure 3B). The results show, as expected, that there was greater variability in the histone peptides with the lowest intensities in all data‐acquisition methods. 75% of the CVs were 20% or below for the DIA60 method. The median CV varied from 10% for DIA60 to 15% for DIA30. In comparing all of the peptide proteoforms together there was a trend to smaller CVs with the DIA60 than the other methods (Figure 3) suggesting a more repeatable quantification method. We saw excellent repeatability in the nano‐LC and time of elution between each run (average CV of elution time < 1%, see Figure S3, supporting information), suggesting that the variability in correctly quantifying the peptides is due to the lower number of MS1 scans and the lower resolution. Furthermore, the chromatography for each peptide was comparable between each data‐acquisition method (Figure 4). Typically, a peptide elutes over a 30 s window, enabling six MS1 scans in DDA120 and up to 20 in DDA30, owing to the shorter cycle time. Furthermore, the DIA60 would result in nine MS1 scans whereas DIA30 would have 14, suggesting that the modest decrease in the CVs is the result of higher resolution rather than the number of MS1 scans.

## 5 CONCLUSIONS

In this study we compared a number of data‐acquisition methods on a Q Exactive HF Orbitrap mass spectrometer for the identification and quantification of histone PTMs. We successfully applied a number of data‐dependent and data‐independent methods to analyze changes in the relative abundance of histone PTMs in CHO cells. We were able to identify 71 histone peptides for histone H3 and H4 and quantified 64 across each of the different acquisition methods.

This study illustrates the versatility of mass spectrometry for the study of changes in relative abundance in histone PTMs. The advantages of DDA for new laboratories mean that the confidence in correctly identifying and quantifying histone PTMs can be achieved with lower resolution MS2 scans when coupled with search engines such as Mascot. Indeed, we demonstrate that the lower resolution DDA30 method was associated with a greater number of PSMs, with equal ability to obtain high ion peptide scores compared to higher resolution methods. However, the advantages of DIA methods over DDA, namely the ability to accurately apportion relative abundances to isobaric co‐eluting peptide proteoforms and that they offer greater flexibility to re‐search data for novel PTMs, outweigh any disadvantages incurred by the technique. In our study we observed increased repeatability in terms of lower CVs afforded by the DIA60 approach when compared with the other data‐acquisition methods. Furthermore, the analysis of DIA data is now made more accessible with the advent of Open Source platforms such as Skyline and dedicated pipelines such as EpiProfile.

## Supporting information


**Figure S1:** Data Acquisition cycle time.
A) The duty cycle time for each data acquisition method is represented. As the resolution is decreased the duty cycle time decreases. B) This shows the number of MS1 and C) MS2 scans for each data acquisition method. As less time is spent on MS2 scans more MS1 scans are undertaken.
**Figure S2:** Average ppm error for each correct identification following Mascot searches. (*n* = 5, mean and standard deviation).
**Figure S3:** Coefficient of Variation of the elution times for the PTMs of KSTGGKAPR peptide identified in EpiProfile.

Table S1: Supporting Information

Table S2: Supporting Information

Table S3: Supporting Information
