# Supplementary material for: Comparison of data‐acquisition methods for the identification and quantification of histone post‐translational modifications on a Q Exactive HF hybrid quadrupole Orbitrap mass spectrometer
Source: Rapid Commun Mass Spectrom. 2019 Apr 26;33(10):897–906. doi: 10.1002/rcm.8401 (PMC6519233; doi:10.1002/rcm.8401)
Supplement: Supplementary file 1 — Figure S1: Data Acquisition cycle time. A) The duty cycle time for each data acquisition method is represented. As the resolution is decreased the duty cycle time decreases. B) This shows the number of MS1 and C) MS2 scans for each data acquisition method. As less time is spent on MS2 scans more MS1 scans are undertaken. Figure S2: Average ppm error for each correct identification following Mascot searches. (n = 5, mean and standard deviation). Figure S3: Coefficient of Variation of the elution times for the PTMs of KSTGGKAPR peptide identified in EpiProfile. [file RCM-33-897-s001.pptx]

## Slide 1
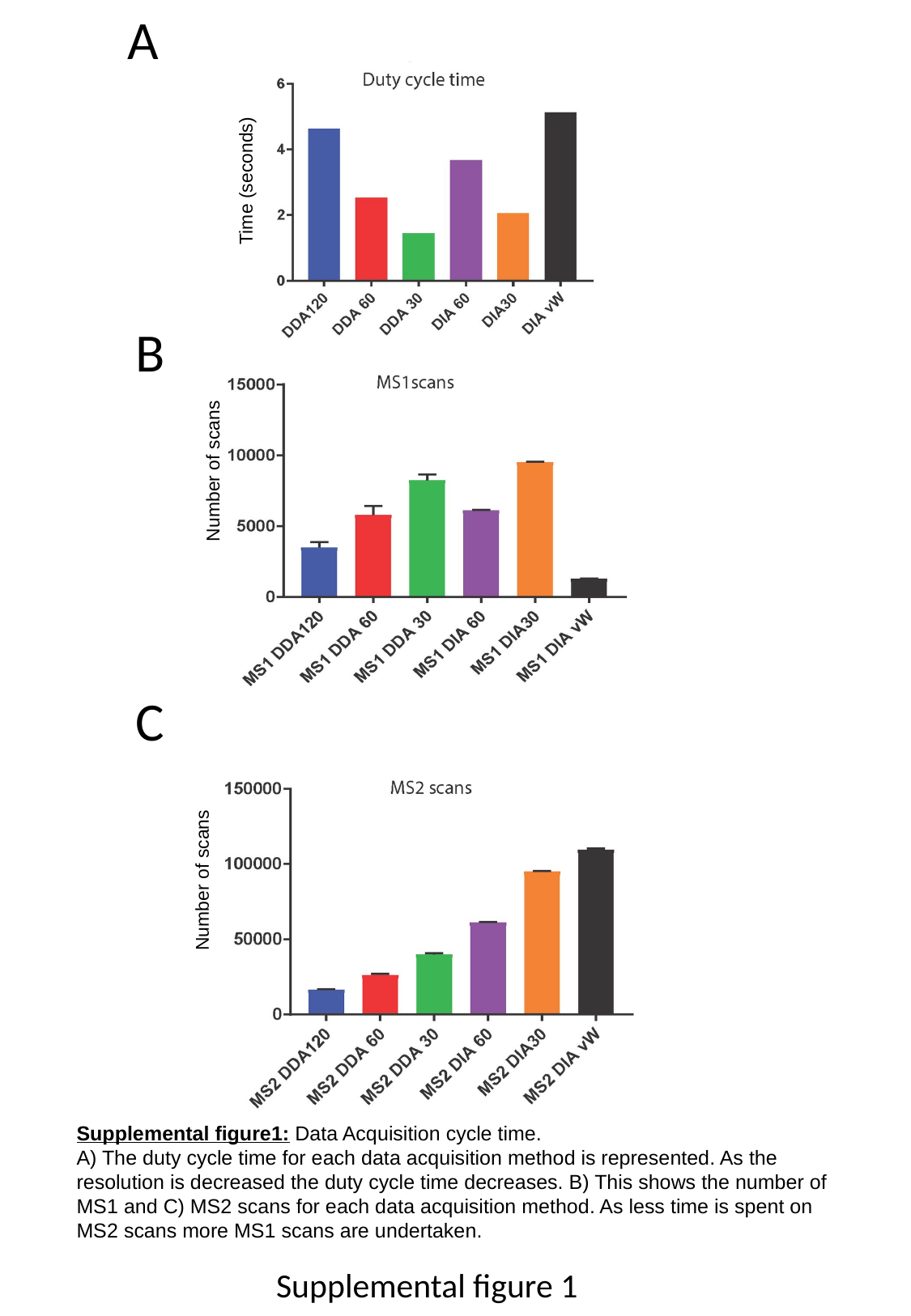

A
Time (seconds)
B
Number of scans
C
Number of scans
Supplemental figure1: Data Acquisition cycle time.
A) The duty cycle time for each data acquisition method is represented. As the resolution is decreased the duty cycle time decreases. B) This shows the number of MS1 and C) MS2 scans for each data acquisition method. As less time is spent on MS2 scans more MS1 scans are undertaken.
Supplemental figure 1

## Slide 2
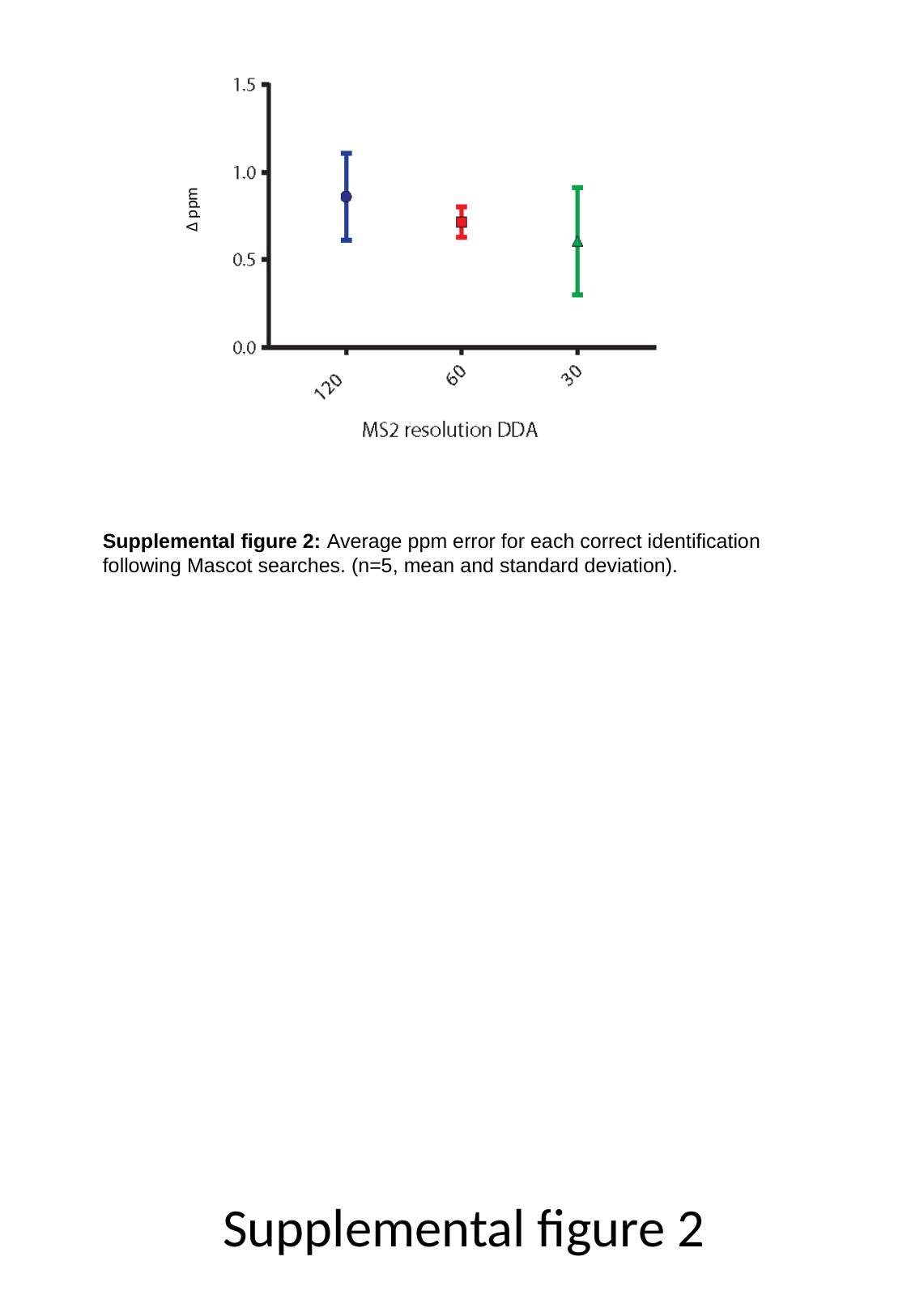

v
Δ ppm
Supplemental figure 2: Average ppm error for each correct identification following Mascot searches. (n=5, mean and standard deviation).
Supplemental figure 2

## Slide 3
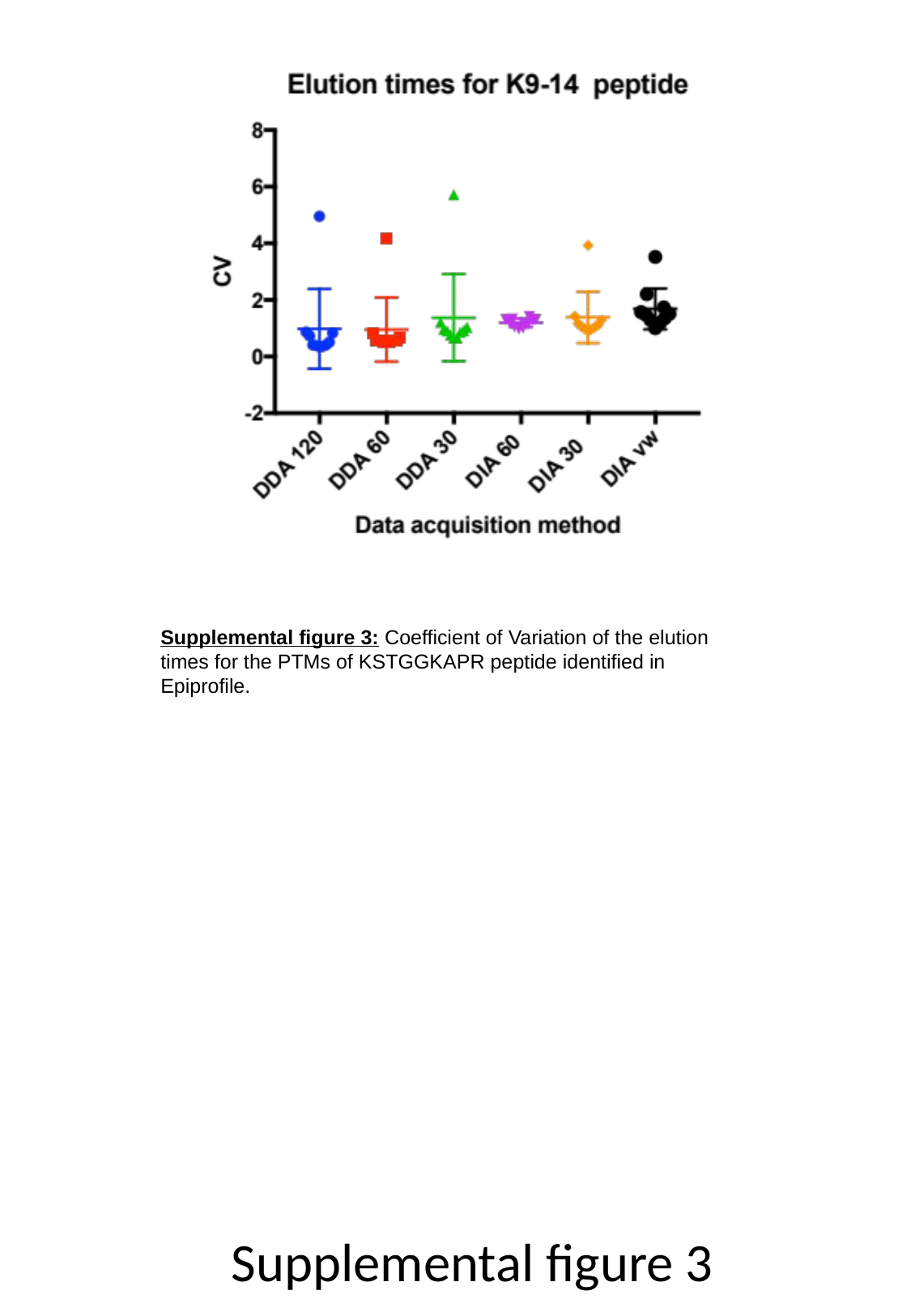

Supplemental figure 3: Coefficient of Variation of the elution times for the PTMs of KSTGGKAPR peptide identified in Epiprofile.
Supplemental figure 3
